# Freeform extended depth-of-focus intraocular lens for more robust performance against physiological variations

**DOI:** 10.1117/1.JBO.31.8.085002

**Published:** 2026-08-25

**Authors:** Julien Charlot, Thierry Lépine, Fannie Castignoles, Vincent Nourrit

**Affiliations:** a IMT Atlantique, Optics Department, Brest, France; b Cristalens Industries, Lannion, France; c LaTIM, INSERM UMR1101, Brest, France; d Jean Monnet University, Laboratoire Hubert Curien, UMR5516, Saint-Etienne, France; e Institut d’Optique Graduate School, Saint-Etienne, France

**Keywords:** extended depth-of-focus, freeform, Zernike

## Abstract

**Significance:**

Extended depth-of-focus (EDOF) intraocular lenses (IOLs) aim to provide improved visual performance over a range of distances, but their clinical outcomes are still sensitive to individual biometric variability. Improving the robustness of EDOF IOL optical performance to these patient-specific variations is critical to enhance visual predictability and patient satisfaction following cataract surgery.

**Aim:**

We aim to determine if freeform surfaces can improve the robustness of EDOF IOLs by maintaining consistent optical performance (refractive error, depth of field) despite individual biometric variations.

**Approach:**

A database of synthetic eyes was adapted to simulate pseudophakic eyes. The post-operative performances (refractive error and depth of field) for three types of EDOF IOLs (L1, L2, L3) were estimated using polychromatic Visual Strehl ratio based on the Optical Transfer Function, with photopic wavelength spectrum for a 3 mm and a 5 mm entrance pupil diameter. L1 is a standard refractive EDOF; L2 is identical to L1 but with a freeform back surface optimized in-eye; L3 is similar to L2 but calculated by a model trained to predict freeform surfaces, using preoperative biometric data (higher-order aberrations of the cornea; effective lens position, calculated from preoperative data; ELP-retina distance; and transverse position of the lens).

**Results:**

The addition of a freeform surface allowed us to significantly reduce the difference between the target refraction and subjective refraction, in polychromatic condition, for small and large pupil sizes while maintaining the depth of field. Performances with L3 were equal to L2.

**Conclusions:**

The use of a freeform surface can significantly reduce the impact of individual biometric variations such as corneal aberrations and transverse IOL positioning on EDOF behavior lenses, for small and large pupil sizes.

## 1 Introduction

Cataract surgery involves replacing the eye’s crystalline lens with an intraocular lens (IOL). This operation corrects a patient’s myopia, hyperopia, and/or astigmatism by selecting the optical power and toricity of the implant. However, as commercially available IOLs are for now a passive optical component, which do not allow for accommodation like the crystalline lens, it necessarily leads to presbyopia. To alleviate this problem, IOLs with complex phase profiles have been proposed to compensate for the loss of accommodation, e.g., with extended depth of field (EDOF) or multifocality.

The performances of EDOF and multifocal IOLs are, however, more sensitive to certain biometric parameters than monofocal IOLs such as IOL decentration and tilt,^1^^,^^2^ photopic pupil size,^3^^,^^4^ and corneal higher-order aberrations.^5^ A solution to better take into account the variety of these parameters in the population would be ideally to provide customized IOLs. Recent technological advances in IOL manufacturing allow for the production of customized IOLs, offering significantly superior performance^6^^,^^7^ compared with a standard aberration-correcting IOL. However, the production of customized IOLs presents several challenges in terms of cost (no economies of scale), scalability (too many specifications), and regulatory and clinical challenges (e.g., FDA approval may require extensive data for each new device, and custom IOLs may require higher skill and consistency from surgeons). Post-surgery tunable IOLs, i.e., IOLs that can be adjusted after implantation,^8^ are a promising innovation, but their capacity to give depth of field (i.e., basically the range of distances for which the image of an object appears sufficiently sharp,^9^ cf. Sec. 2.4) is for now limited.

In parallel, progresses made in the design and manufacturing of nonrotationally symmetric geometries for other industries (aerospace, illumination, etc.) have shown how freeform optics can allow us to simultaneously correct multiple aberrations across wide fields of view and push optical specifications beyond the limits of traditional lenses.^10^ As the reduced performances of EDOF IOLs in eyes with atypical characteristics are attributable to complex phase profiles (e.g., resulting from the interaction of the EDOF profile with higher-order corneal aberrations, lens tilts, and/or decentrations), the adoption of a freeform profile could potentially offer a corrective solution. We denote here by “atypical characteristics”: ocular parameter values that fall outside the normal range but also parameters that are not usually considered during IOL design such as higher-order ocular aberrations or lens transverse position.

In this context, the aim of the study was to take advantage of recent advances in freeform optics to investigate if the use of a freeform profile could allow the design of a more robust EDOF IOL, i.e., guaranteeing good optical performance (refractive error and depth of field) for a greater majority of the population.

## 2 Method

To study the benefit of a freeform EDOF IOL compared with a classic one for a range of eyes covering the variety of physiological parameter variations in the population, a number of steps have to be carried out.

First, we need to define the parameters taken into account and a database of eyes large enough to reflect the variation of these parameters in the population. To do this, we chose to start with a pre-op eye database (Sec. 2.1), which we modified to obtain a database of post-op eyes (Sec. 2.2). These post-op eyes were corrected by a classic EDOF IOL presented in Sec. 2.3. In Sec. 2.4, we define the metrics that will be used to optimize the EDOF IOL with a freeform profile and compare it with the classic EDOF solution. Finally, the freeform EDOF is presented in Sec. 2.5.

### 2.1 Preoperative Database

Many biometric parameters can influence the optical performances of implants aimed at improving depth of field such as corneal curvature radii, corneal aberrations, anterior chamber length or lens tilt and decentration.^5^ Although many studies have examined the distribution of each parameter in the population individually, there is no database in which all these parameters are simultaneously documented for a large number of subjects. To generate a realistic eye, all these parameters cannot be assumed to vary independently. Contradictory results have been obtained on some parameters (e.g., Porter et al. concluded that there was virtually no correlation between the Zernike modes of the eye,^11^ disagreeing with Thibos, Hong et al. ^12^), but it is clear that some parameters are correlated (e.g., corneal aberrations and axial length^13^). We therefore used the database proposed by Rozema et al.,^14^ which is distributed under a CC-BY-NC-ND 4.0 International License. The dataset was used as provided, without redistribution of modified versions. It consists of 1000 synthetic eyes (SyntEyes), based on the Navarro eye model and generated from measurements obtained from 312 normal eyes. It considers a very large number of physiological parameters, including anterior and posterior corneal aberrations, central corneal thickness (CCT), anterior chamber depth (ACD), lens thickness (LT), tilt and decentration of the pupil and the lens, axial length (AL), etc. The associated statistics are presented in Table 1.

**Table 1:** Ocular parameters of the SyntEyes database. Pupil and lens shift and decentering are relative to the keratometric axis, as defined in the database. The corneal aberrations are the aberrations of the total cornea (anterior and posterior) and are expressed for a 6 mm entrance pupil diameter.

Parameter	Mean	Std	Min	Max
CCT (mm)	0.548	0.031	0.456	0.659
ACD (mm)	2.901	0.39	1.726	4.395
LT (mm)	4.062	0.379	2.797	5.22
AL (mm)	24.04	1.08	21.18	27.71
Pupil and lens decentering (mm)	0.21	0.101	0.024	0.661
Pupil and lens shifting (deg)	3.833	1.065	0.736	6.991
Corneal aberrations				
Astigmatism (μm)	0.631	0.383	0.045	2.166
Coma (μm)	0.263	0.135	0.009	0.803
S.A (μm)	0.232	0.068	0.035	0.435
Trefoil (μm)	0.051	0.026	0.002	0.158
Sec. astigmatism (μm)	0.049	0.025	0.001	0.148
Quadrafoil (μm)	0.041	0.017	0.001	0.125

CCT, central corneal thickness; ACD, anterior chamber depth; LT , lens thickness; AL, axial length; S.A, spherical aberration.

### 2.2 Postoperative Database

To obtain a database of post-op eyes on which to study the benefits of a freeform implant, we simulated pseudophakic eyes using ANSYS Zemax by replacing the lens with an IOL. Predicting the exact outcome of an implant and changes in the physiological parameters of the eye before cataract surgery can be challenging due to individual variations in anatomy and healing responses. Preoperative assessments and advanced imaging techniques help improve accuracy, but some uncertainty remains. A few assumptions were therefore necessary.

With regard to corneal aberrations, surgery can induce a modification of the anterior surface of the cornea, inducing spherical aberration, astigmatism, coma, and trefoil, which may depend on the surgeon’s incision axis.^15^ As no model was found to predict these changes, and as post-op corneal aberrations are of the same order of magnitude as pre-op aberrations for small incision sizes,^16^^,^^17^ we decided not to modify these corneal aberrations in our cataract simulation.

To calculate the longitudinal position of the implant, we used the Haigis formula (with $a_{0}$, $a_{1}$, and $a_{2}$ constants optimized by the manufacturer, here Cristalens Industrie (Lannion, France):

$$\mathrm{ELP}=a_{0}+a_{1}\times {\mathrm{ACD}}_{\text{pr⏧op}}+a_{2}\times \mathrm{AL},$$

with ELP, the effective lens position of the IOL; ACD, the anterior chamber depth; and AL, the axial length of the eye. Constants used are $a_{0}=0.088$, $a_{1}=0.233$, $a_{2}=0.2$.

With regard to the longitudinal position of the pupil, the pupil and the anterior surface of the lens are almost in the same plane before surgery. This is not the case afterward.^18^ As we were unable to find a formula for calculating the distance between the iris and the anterior surface of the IOL, we placed the pupil on the anterior surface of the IOL and fixed the size of the entrance pupil of the optical system (which is the size of the pupil seen by the surgeon).^19^

For the transverse position of the different optical surfaces of each eye, all the data of the database were measured with the keratometric axis as a reference axis. Thus, the keratometric axis is the reference axis in this study. The anterior and posterior surfaces of the cornea are positioned relative to this axis using the nonzero values of the Zernike tilt coefficient. The IOL tilt and decentration were kept equal to the values of the crystalline lens, relative to the keratometric axis defined in the database. The horizontal decentration of the IOL is 0.11±0.04  mm([−0.01,0.22]), the vertical decentration was  −0.07±0.19  mm([−0.63,0.53]), the horizontal tilt was 3.71±1.09  deg ([−0.19,6.95]), and the vertical tilt was 0.08±0.93  deg ([−3.22,3.17]). These values are expressed in the IOL plane, after implantation at the ELP position.

### 2.3 EDOF IOL (L1)

To our knowledge, complete technical data of any commercial EDOF IOL are not available. We therefore defined a database of generic EDOF implants (denoted L1) to be used to correct the post-op eyes defined in Sec. 2.2.

The optics of an EDOF IOL can be seen as comprising two parts: a “correction” part and a “performance” part. The correction part comprises the “spherical” power of the implant (correcting myopia and hyperopia), its toricity (correcting astigmatism), and its aspherization (or conicity) in the case of aspheric IOLs, designed to totally or partially correct corneal spherical aberration.

For the “correction” part of our EDOF, we used a lens made in hydrophobic acrylic material (n=1.54), with a front surface that is aspherical and a back surface that is biconic, with a rear principal spherical radius equal to the front one. We optimized this lens in a Liou-Brenman eye model to achieve a range of powers and toricity, respectively, from 10D to 35D (0.5D steps) and from 0D to 4.5D (0.75D step), keeping the central thickness between 0.4 and 0.8 mm. The aspheric coefficient of the front surface has been optimized to fully correct a corneal spherical aberration equal to 0.28  μm (for a 5.15 mm diameter pupil, placed at the front surface of the IOL, as defined by the standards ISO 11979-2).

Regarding the “performance” part that provides the extended depth of field, it can be implemented in different ways, e.g., with diffraction rings, a power variation profile, or spherical aberration.^20^ In our case, it consists of a central zone, 2 mm in diameter on the front surface where the higher-order spherical aberration coefficients ($Z_{4}^{0}$ to $Z_{12}^{0}$) are optimized for a 3 mm pupil to ensure that the polychromatic modulation transfer function is higher than 0.2 (corresponding to 0.2 LogMAR, 20/32 on the Snellen scale or 0.63 on the decimal scale ^21^) over 1.75D from the distance vision peak. The average values of the standard Zernike coefficients are given in Table 2.

**Table 2:** Standard Zernike spherical aberration coefficients induced by the EDOF IOL on an L&B model entrance pupil plane.

$Z_{4}^{0}$	−0.10±0.03 μm
$Z_{6}^{0}$	−0.01±0.03 μm
$Z_{8}^{0}$	0.05±0.02 μm
$Z_{10}^{0}$	0.0±0.02 μm
$Z_{12}^{0}$	−0.02±0.01 μm

There are various formulas for calculating the power and toricity of an IOL needed to correct a patient’s defocus and astigmatism. However, as we are working with digital eyes here, we wanted to correct for objective refraction, which consists of making the Zernike coefficients of defocus and astigmatism ($Z_{2}^{0},Z_{2}^{2}$, and $Z_{2}^{-2}$) zero. Therefore, to find in our set of EDOF IOL which one to use to correct the post-op SyntEye, we used the following method. The lens was replaced by a lens similar to the correction part defined above. Radii of curvature (front surface and the 2 radii of the posterior surface) and the IOL tilt along the longitudinal axis were optimized to cancel out these Zernike coefficients over a 5 mm EPD. The calculated power and toricity of the IOL were then, respectively, rounded to the nearest 0.5D and 0.75D to choose the adequate EDOF IOL.

### 2.4 Metric Used to Assess Post-Op Vision Quality and Depth of Field Performance

Numerous metrics have been proposed to relate visual quality to ocular aberrations. Among them, the Visual Strehl ratio based on the Optical Transfer Function (VSOTF) correlates best with the number of incorrect letters read by a patient after inducing aberrations.^22^ However, as the SyntEye simulated in this study contains asymmetric aberrations such as coma, secondary astigmatism, and others, we used an enhanced version of this metric, proposed by Iskander in 2006,^23^ taking into account the impact of these aberrations. In addition, as IOLs can have different chromatic behaviors, depending on their Abbe number, and whether they are refractive or diffractive, we used in this study the polychromatic version of ${\mathrm{VSOTF}}_{a}$, defined as

VSOTFaPoly=∬060CSF(fx,fy)|Re{OTFPoly}|dfxdfy∬060CSF(fx,fy)OTFDLPolydfxdfy,

where $f_{x},f_{y}$ are the spatial frequencies in cycles per degree and CSF is the contrast sensitivity function as defined by Campbell and Green,^24^

$$\mathrm{CSF}\left(f\right)=2.6\left(0.00192+0.114f\right)\exp\left[-{\left(0.114f\right)}^{1.1}\right].$$

${\mathrm{OTF}}_{\mathrm{poly}}$ is the optical transfer function calculated from the polychromatic ${\mathrm{PSF}}_{\mathrm{poly}}$ defined as^25^

$${\mathrm{PSF}}_{\mathrm{poly}}=\int S\left(\lambda \right)\mathrm{PSF}\left(\lambda \right)d\lambda ,$$

where $S_{\lambda }$ is the spectral luminance weight. $\mathrm{OT}{F_{\mathrm{DL}}}_{\mathrm{Poly}}$ represents the polychromatic optical transfer function for a diffraction-limited eye (3 mm pupil diameter).

Working in polychromatic conditions imposes conditions on the spectrum of the source used and on the definition of the chromatic behavior of the materials used in the optical system. For the light source, we used the photopic source predefined in the ANSYS Zemax software. It is discretized into five wavelengths, satisfying the minimum sampling criterion of 50 nm defined in the study,^25^ such as

$$\begin{array}{c} S{\left(\lambda \right)}_{\mathrm{phot}}=\left\{ \begin{array}{l} 0.091\lambda =470\;\mathrm{nm}\\ 0.503\lambda =510\;\mathrm{nm}\\ 1\lambda =555\;\mathrm{nm}\\ 0.503\lambda =610\;\mathrm{nm}\\ 0.107\lambda =650\;\mathrm{nm} \end{array}\right. \end{array}.$$

For the chromatic behavior of the materials making up the eye, we used Atchison’s equations defined in his 2005 study.^26^ The material chosen for IOLs in this study is the premium IOL’s material of the manufacturer, which is a hydrophobic acrylic material with a refractive index of 1.54.

In the rest of the document, to simplify the writing, we chose to refer to $\mathrm{VSOT}{F_{a}}_{\mathrm{Poly}}$ simply as ${\mathrm{VSOTF}}_{\mathrm{poly}}$.

Performances of an EDOF lens are often assessed in terms of post-operative refractive error and depth of field. Refractive error is a clinical concept that represents the power in diopters that needs to be added to the patient’s vision to make him emmetropic. It is therefore not possible to estimate it here, as we have numerically simulated a cataract operation. We thus choose to estimate the post refractive error by calculating the difference between the position of the ${\mathrm{VSOTF}}_{\mathrm{poly}}$ distance vision peak (defined as the first maximum value of the through focus VSOTF curves) and the retinal plane.^27^

With regard to the depth of field, it is generally quantified using visual acuity and a 0.2 logMAR drop from maximum acuity. Considering that a visual acuity value of 0.2 logMar corresponds approximately to a monochromatic VSOTF value of 0.12,^28^ and considering chromatic behavior reduces the VSOTF value approximately by 16%, ^29^ we took a criterion of ${\mathrm{VSOTF}}_{\mathrm{poly}}$ better than 0.1 to quantify the depth of field of our post-op SyntEyes. Depth of field was calculated before and after freeform surface for a 3 and 5 mm entrance pupil diameter, with the photopic wavelength spectrum.

### 2.5 Model Optimization of Freeform Surfaces (L2)

After replacing the crystalline lens with a basic EDOF IOL (denoted L1), the posterior surface of the EDOF IOL was replaced with a freeform surface to correct for the monochromatic aberrations (at 550 nm) of each SyntEye. We refer to this freeform IOL as (L2). Various bases exist for representing a freeform surface (e.g., XY, Nurbs, Zernike^30^). We chose to define this surface in Ansys Optics Studio as a standard Zernike surface (SZERNSAG), thus using standard Zernike polynomials.

To optimize the freeform surface, the merit function was defined to make the standard Zernike coefficients of the orders 2, 3, and 4 of the eye equal to 0, by setting the same coefficients on the freeform surface as variables (with the exception of the defocus coefficient $Z_{2}^{0}$, for which the radius of curvature was set as a variable). In other words, optimization was stopped at the 15th standard Zernike coefficient and excepting the piston and tilt’s coefficient. These 15 first coefficients may not theoretically capture all clinically significant higher-order aberrations (particularly if significant aberrations are present^31^), but we chose not to consider other higher coefficients for sake of simplicity and because according to the literature, an eye model can be considered correctly corrected when the monochromatic Strehl ratio at 545 nm reaches 0.82 (Marechal Criterion, which was achieved in our case, in 98% of eyes for a 5 mm pupil). In view of these good results, we considered that correcting higher orders would unnecessarily complexify the freeform surface for limited benefits.

To maintain the depth of field, the EDOF zone (Sec. 2.3) was not considered during optimization (otherwise correcting the Zernike would have canceled this DOF). The mean values of the Strehl ratio for the 1000 eyes after optimization of this freeform surface are 0.930±0.043. The new freeform IOLs, therefore, fully correct the aberrations of the simulated eyes (despite possibly strong tilt and decentration of the lens). We then added back the EDOF zone to the center of the front surface of the freeform IOLs to achieve the desired depth-of-field performance.

### 2.6 Model to Generate Freeform Surfaces from Biometric Data (L3)

Practically, particularly from a manufacturer’s point of view, these freeform surfaces would be ideally generated using preoperative or highly predictable post-operative biometric parameters, and a limited range of freeform surfaces would be required to address patients’ needs.

**Fig. 1:**
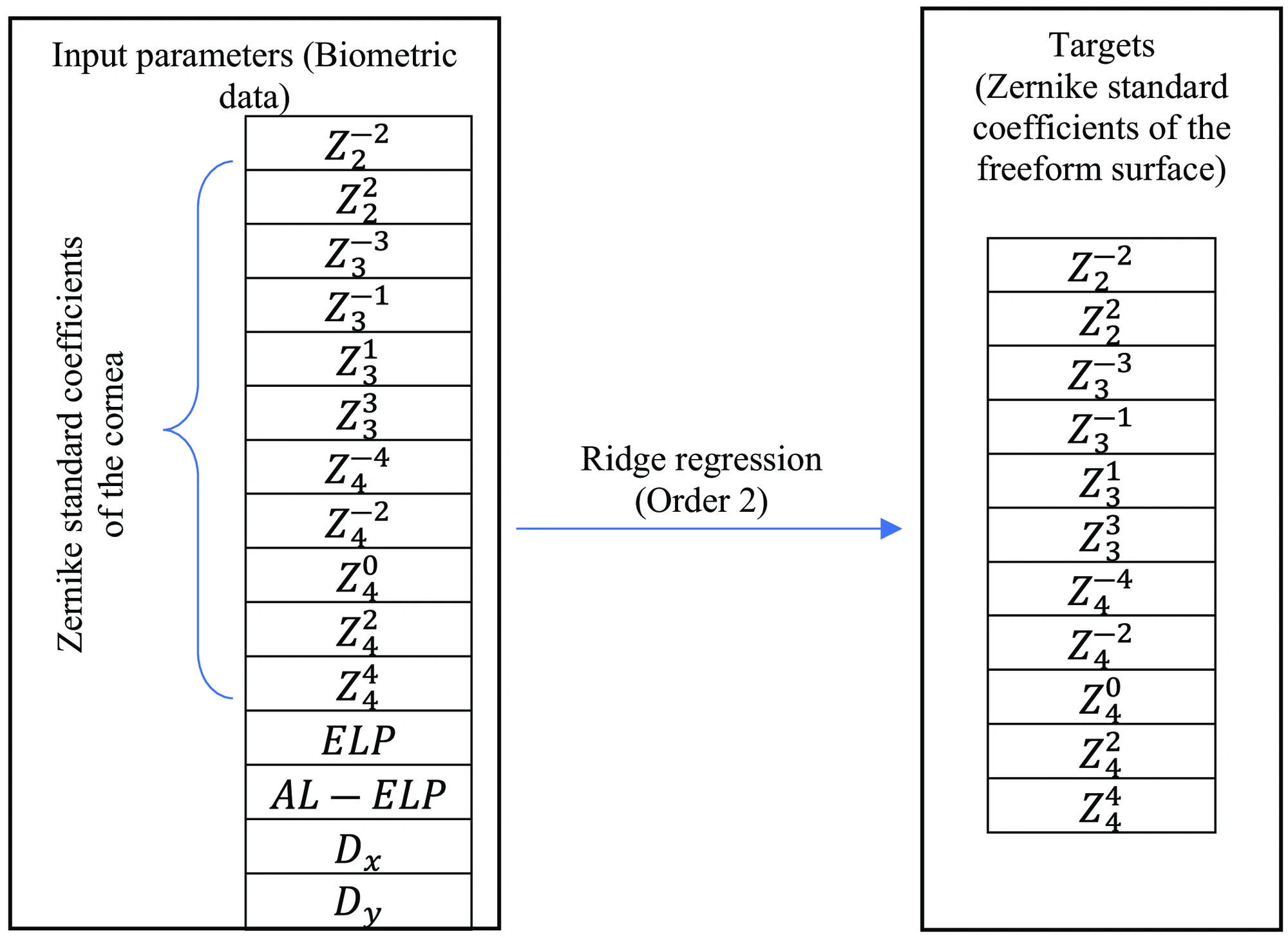
Input parameters and targets of the trained model, aiming at the generation of the freeform surfaces. ELP, effective lens position; AL, axial length; $D_{x},D_{y}$, decentering of the IOL relative to the visual axis.

We therefore assessed if it was possible to predict the Zernike coefficients of the freeform surface from preoperative biometrics parameters, which are the higher-order aberrations (HOA) of the cornea, the effective lens position (ELP) as defined in Sec. 2.2 calculated from preoperative data, the distance between the ELP and the retina, and the transverse position of the crystalline lens. These parameters are depicted in Fig. 1. We denote the EDOF lens calculated with these new Zernike coefficients (L3). To predict the standard Zernike coefficients of the freeform surfaces, we employed Ridge regression—a form of multilinear regression—implemented using the scikit-learn library in Python.^31^^,^^32^ A second-order model was used, allowing for the inclusion of cross-terms between variables. The model was trained on data from 500 SyntEyes, and its performance was evaluated on a separate set of 500 SyntEyes.

## 3 Results

### 3.1 Refractive Error

**Fig. 2:**
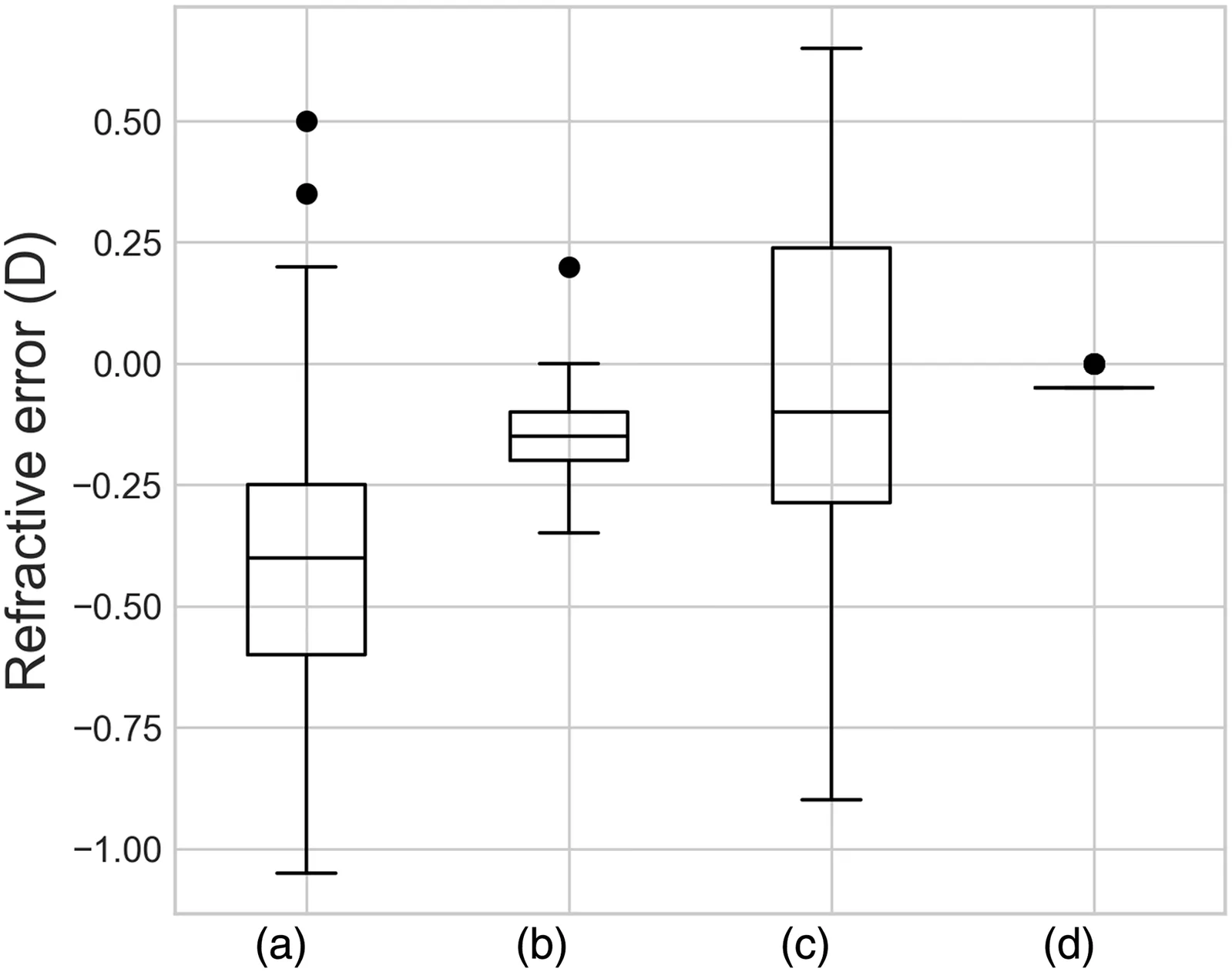
Refractive error for (a) 3 mm pupil, without freeform surface, (b) 3 mm pupil, with freeform surface, (c) 5 mm pupil without freeform surface, and (d) 5 mm pupil with freeform surface. Black dots are outliers.

For the classic EDOF IOL, the positions of the distance vision ${\mathrm{VSOTF}}_{\mathrm{poly}}$ peaks are at −0.39±0.32 D for small pupil size (Fig. 2). This is similar to the average difference between the aimed refraction and the subjective post-op refraction (−0.39±0.42 D) reported in the EUREQUO annual report (2022) and based on 218,421 cataract surgeries. After optimization with the freeform surface, the positions of the distance vision VSOTF peaks are reduced to  −0.15±0.09 D. As the EDOF zone is centrally located within the implant, its optical contribution is maximized under small pupil conditions. With conventional EDOF designs, visual outcomes arise from the interaction between this central zone, potentially decentered and tilted relative to both the visual axis and the pupil, and existing corneal aberrations. These combined effects lead to an average myopic shift of approximately −0.39  D, which is not necessarily disadvantageous from a clinical perspective. Indeed, targeting a mild myopization of −0.50 D to −0.75 D in one eye may be intentionally pursued to enhance binocular depth of field. However, to achieve better control over this effect, it is preferable to aim for a theoretical refractive error as close as possible to emmetropia (0.00 D), thereby allowing the surgeon to decide whether to induce myopization. The introduction of a freeform intraocular lens (IOL) reduces the average myopic shift to −0.15 D. In addition, refractive variability is significantly reduced, with the standard deviation decreasing from 0.32 D to 0.09 D, improving the predictability of the location of the best visual acuity. The remaining variability observed with the freeform IOL is mostly due to residual decentration and tilt of the EDOF component, which are not corrected by the freeform surface.

Under large pupil conditions, the refractive error decreases from −0.05±0.32 D with conventional EDOF to −0.04±0.02 D with the freeform design. In this context, corneal aberrations exert a greater influence than the EDOF component, accounting for the substantial reduction in variability. The reduction in mean values due to the freeform design in both conditions (Fig. 2) shows that a freeform IOL could reduce the gap between the desired refraction and the subjective post-op refraction, enabling improved prediction of post-op refractive errors. This gap is due to two factors. First, L1 is optimized to minimize defocus and astigmatism (cf. Sec. 4.3), and therefore, the presence of higher aberrations (e.g., spherical aberration of the cornea) can shift the position of the VSOTF peak. Furthermore, to be representative of implants on the market, the calculated power and toricity of the IOL were rounded to the nearest 0.5D and 0.75D, respectively. In addition, the reduction in standard deviations shows that this freeform surface could also considerably reduce refractive error variations due to individual corneal aberrations and IOL positioning.

Furthermore, the absolute difference between the location of the ${\mathrm{VSOTF}}_{\mathrm{poly}}$ peak for small and large pupil sizes passed from 0.34±0.47 D, to 0.11±0.09 D after optimization. The reduction of the standard deviation shows that thanks to a freeform surface, visual outcomes are less pupil dependent.

### 3.2 Depth of Field

As the optimization of the freeform surfaces were carried out without considering the EDOF profile, it is necessary to assess the potential impact of the freeform surface on the depth of field.

**Fig. 3:**
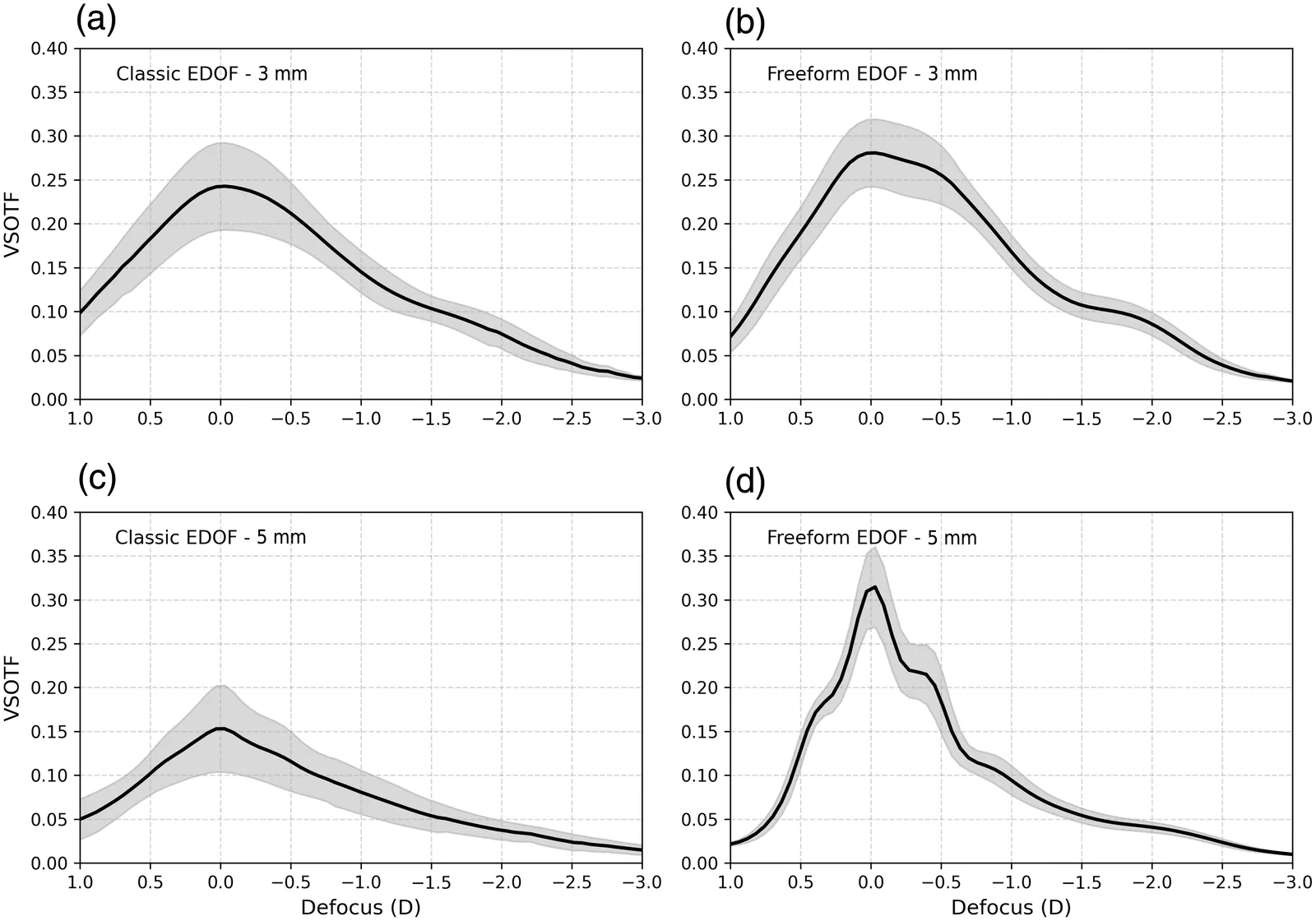
Through focus $\mathrm{VSOT}F_{\mathrm{poly}}$ of the SyntEyes with a classical EDOF and the freeform EDOF for each condition: (a) classic EDOF with a 3 mm pupil, (b) freeform EDOF with a 3 mm pupil, (c) classic EDOF with a 5 mm pupil, and (d) freeform EDOF with a 5 mm pupil.

**Fig. 4:**
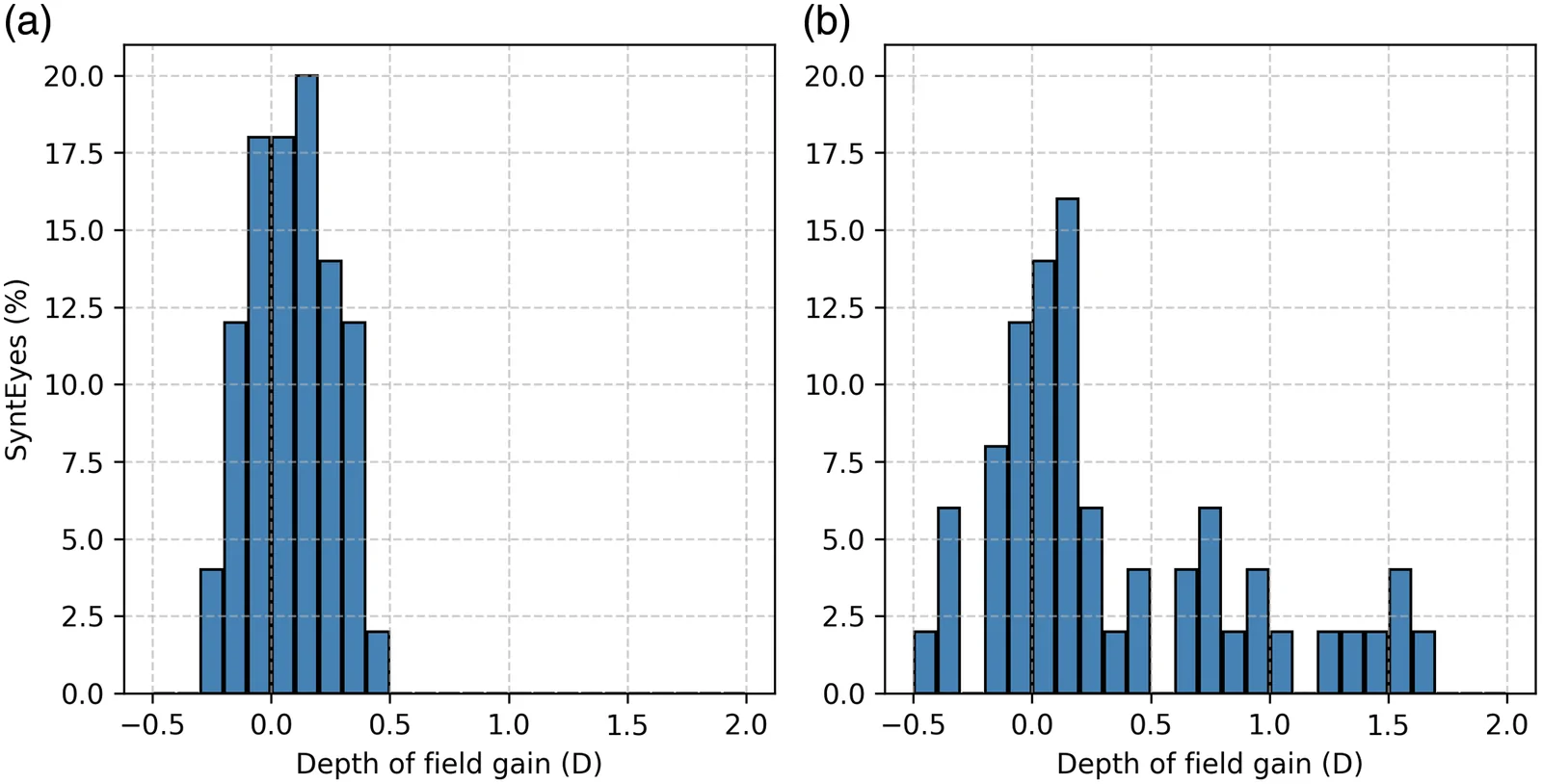
Depth of field gain with use of a freeform surface with (a) a 3 mm pupil and (b) a 5 mm pupil.

To better visualize how the ${\mathrm{VSOTF}}_{\mathrm{poly}}$ changes in different conditions [classic EDOF (L1) versus freeform EDOF (L2), for small and large pupil size], the through focus ${\mathrm{VSOTF}}_{\mathrm{poly}}$ curves, once recentered on the distance vision peak, were averaged and plotted in Fig. 3. The change in depth of field for small pupil between the classic and freeform EDOF is 0.061±0.165 D. The freeform surfaces, designed to correct the aberrations of the simulated eyes, therefore have on average a little impact on the depth of field provided with small pupil size. If we look more precisely at these results (Fig. 4), the DOF will be increased for ∼66% of the SyntEyes (up to 0.5D) and reduced for 34% of them (up to 0.3D). For 68% of the SyntEyes, the DOF variation is between ±0.2 D. The maximum value of the VSOTF curves passed from 0.248±0.049 to 0.278±0.039.

For a large pupil size, the freeform surface improves the depth of field in average (0.33±0.55 D) and the maximum value of the VSOTF curve passing from 0.15±0.05 to 0.32±0.04. 28% of SyntEyes lose some DOF (20% ∈[−0.20,0] D and 8% ∈[−0.50,−0,20] D) when 72% of them have their depth of field increased (up to 1.7D) (Fig. 4).

### 3.3 Accuracy of Freeform Surface Generated with Preoperative Data (L3)

The results from Secs. 3.1 and 3.2 suggest that the use of a freeform surface can improve the performance of an EDOF IOL in response to fluctuations in physiological parameters (including pupil size). However, these results are based on a “post-op” optimization for each eye. To assess the model’s accuracy, the predicted Zernike coefficients were linearly fitted against the corresponding coefficients calculated by Zemax for the test set. The results, summarized in Table 3, show that all coefficients of determination ($R^{2}$) exceed 0.96, with the slopes of the linear fits being nearly equal to 1, indicating strong agreement between the predicted and reference values.

**Table 3:** Performance of the linear regression between the standard Zernike coefficients calculated by Zemax and by our model, for each coefficient.

Zernike coefficients	Coefficient of determination ($R^{2}$) between the coefficient calculated by Zemax and predicted by our model	Slope of the fit
$Z_{2}^{-2}$	0,98	0.99
$Z_{2}^{2}$	1	0.99
$Z_{3}^{-3}$	0.99	0.98
$Z_{3}^{-1}$	0.97	0.97
$Z_{3}^{1}$	0.96	0.97
$Z_{3}^{3}$	0.98	0.98
$Z_{4}^{-4}$	1	1
$Z_{4}^{-2}$	1	0.99
$Z_{4}^{0}$	0.99	0.99
$Z_{4}^{2}$	0.99	0.99
$Z_{4}^{4}$	1	1

**Fig. 5:**
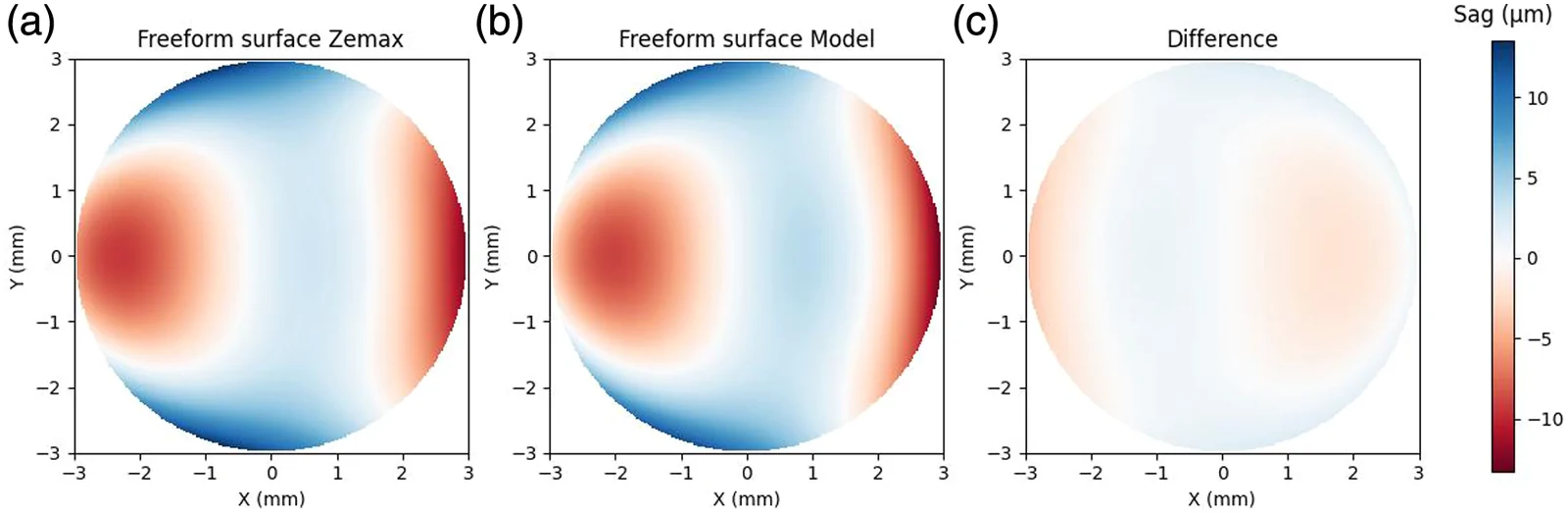
Freeform surfaces aiming for the correction of the HOA of one SyntEye, calculated by Zemax (a), by our model (b), and the difference between them (c).

**Fig. 6:**
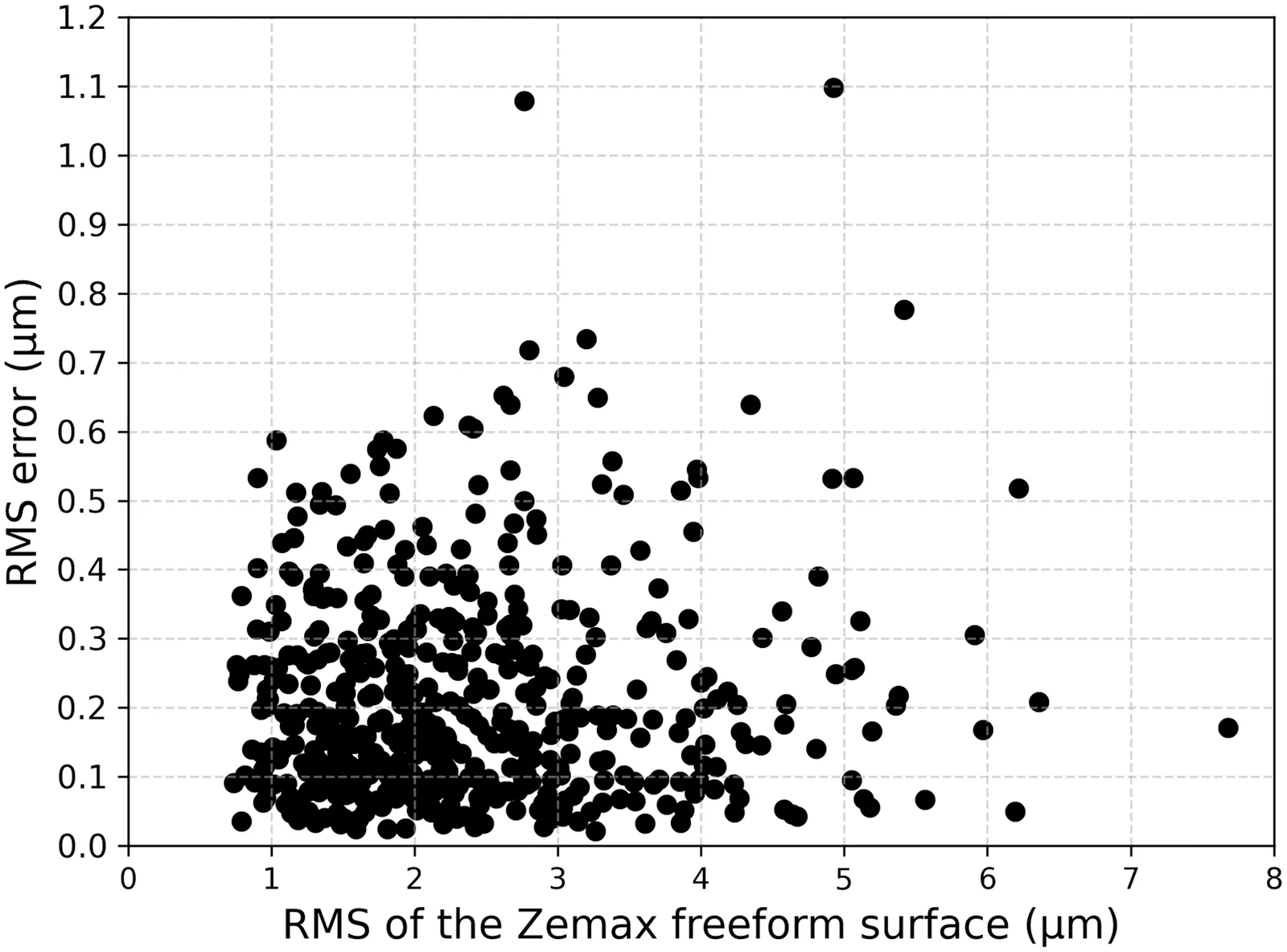
RMS of the difference between the Zemax freeform and our model freeform surface, depending on the RMS of the Zemax freeform.

Although our model predicts each Zernike coefficient with high accuracy, prediction errors persist. As the generation of the freeform surface relies on 15 parameters, even small errors in individual predictions can accumulate, potentially leading to a noticeable deviation between the intended surface and the one produced by our model. To quantify this effect, we computed the difference between the targeted freeform surfaces (generated by Zemax) and those reconstructed using our predicted coefficients. We then calculated the root mean square (RMS) of these differences and compared it with the RMS of the target surfaces. An illustration of these surfaces is shown in Fig. 5, and the results of these deviations are plotted in Fig. 6. The mean RMS of the difference is 0.216±0.158  μm, which represents a mean variation of 10.98±9.32% of the RMS of the desired freeform surfaces.

**Fig. 7:**
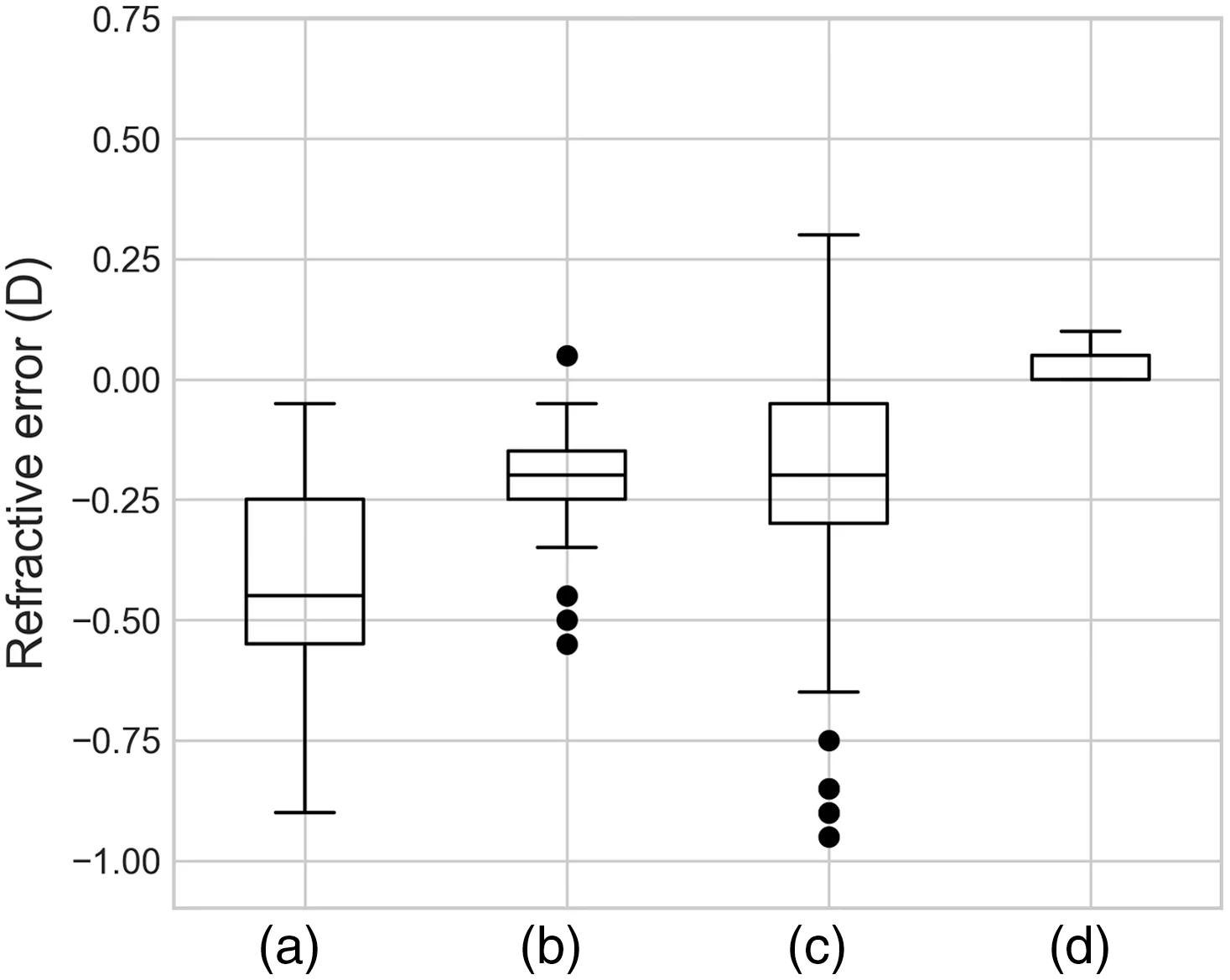
Refractive error of SyntEyes corrected with (a) classic EDOF with a 3 mm pupil, (b) EDOF with a freeform surface generated by our model with a 3 mm pupil, (c) classic EDOF with a 5 mm pupil, (d) EDOF with a freeform surface generated by our model with a 5 mm pupil.

**Fig. 8:**
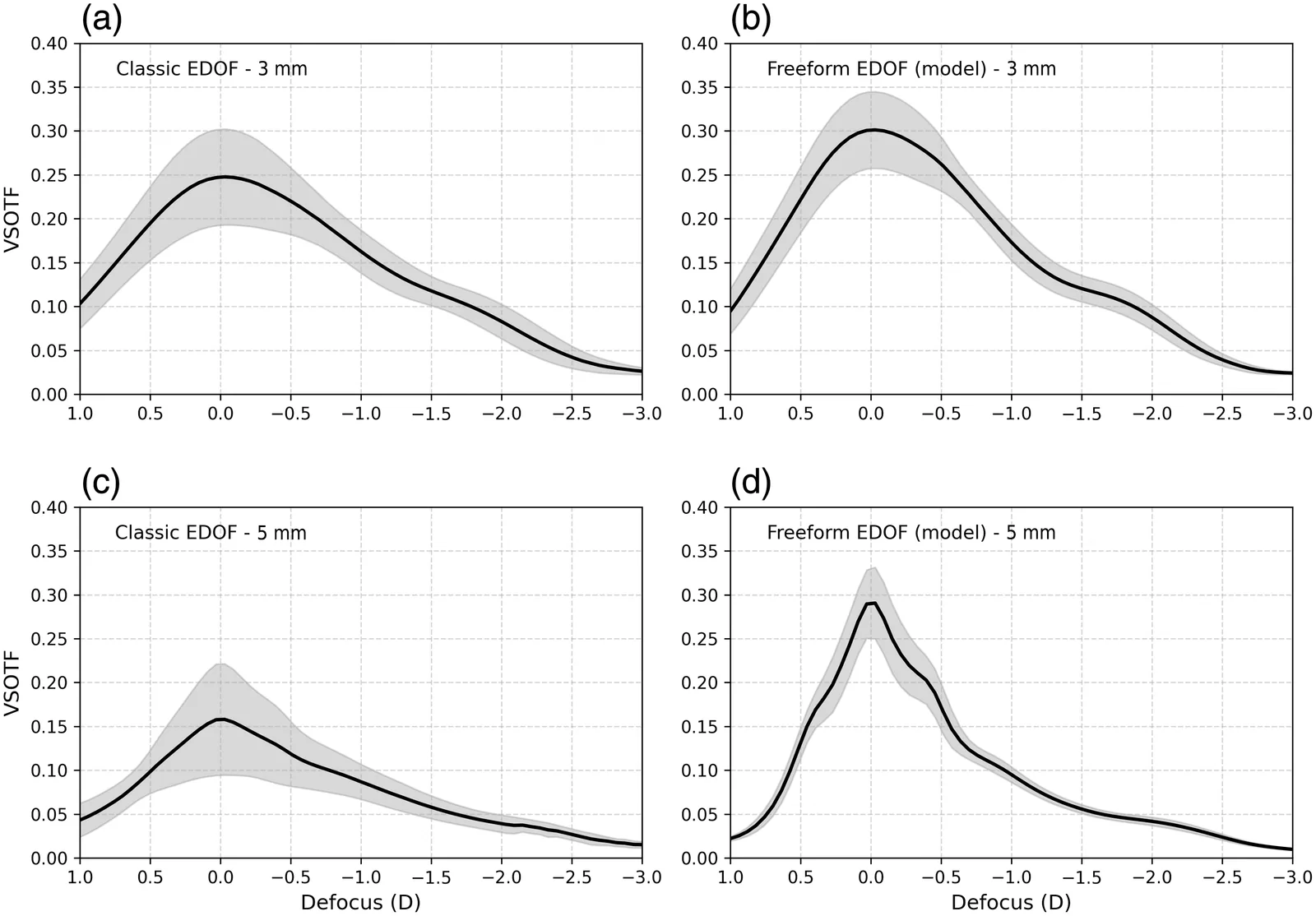
Through focus $\mathrm{VSOT}F_{\mathrm{poly}}$ of the SyntEyes with a classical EDOF and the freeform EDOF for each condition; (a) classic EDOF with a 3 mm pupil, (b) freeform EDOF generated by our model with a 3 mm pupil, (c) classic EDOF with a 5 mm pupil, (d) freeform EDOF generated by our model with a 5 mm pupil.

As there are still some errors, equal to ∼11% of the target freeform surfaces, we wanted to validate the performance of the freeform surfaces generated by our model. We then simulated their performances with the generated freeform EDOF IOLs in the same condition (photopic waveband, with 3 and 5 mm pupil) as defined in Sec. 2.4. Comparisons between the classic EDOF (L1) and freeform EDOF generated by our model (L3) are plotted in Fig. 7 for refractive errors and Fig. 8 for ${\mathrm{VSOTF}}_{\mathrm{poly}}$ curves.

For a 5 mm pupil, the peak values of the $\mathrm{VSOT}{F_{a}}_{\mathrm{poly}}$ of the SyntEyes corrected with EDOF IOL with a freeform surface generated by our model (L3) are 0.295±0.040, against 0.318±0.046 with freeform surfaces calculated with Zemax (L2), and 0.154±0.05 with a classic EDOF (L1). The freeform surfaces generated by our model permit to significantly improve the peak values of the ${\mathrm{VSOTF}}_{\mathrm{poly}}$, which theoretically corresponds to an improvement of the corrected distance visual acuity (CDVA). The error on the freeform surfaces generated by our model induced a mean reduction of the peak value equal to 0.024, corresponding to 8% loss compared with the IOL with a freeform surface generated by Zemax. For a 3 mm pupil size, peak values are, respectively, 0.298±0.048 (L3), 0.278±0.039 (L2), and 0.248±0.049 (L1). As L3 has better results than L2 (∼7%), we have supposed that the error generated by our model on freeform surfaces interacts with the performance part of the optic (EDOF), slightly improving the peak values of the $\mathrm{VSOT}{F_{a}}_{\mathrm{poly}}$ and slightly decreasing the depth of field.

The refractive errors for L3 are, respectively,  −0.21±0.12 D and 0.03±0.03 D for small and large pupil size, against  −0.15±0.09 D and −0.04±0.02 D for L2, and  −0.39±0.32 D and  −0.05±0.32 D for L1. The freeform surfaces generated by the model therefore produce similar results to those generated by Zemax in terms of refractive error, both in mean and standard deviation values.

**Fig. 9:**
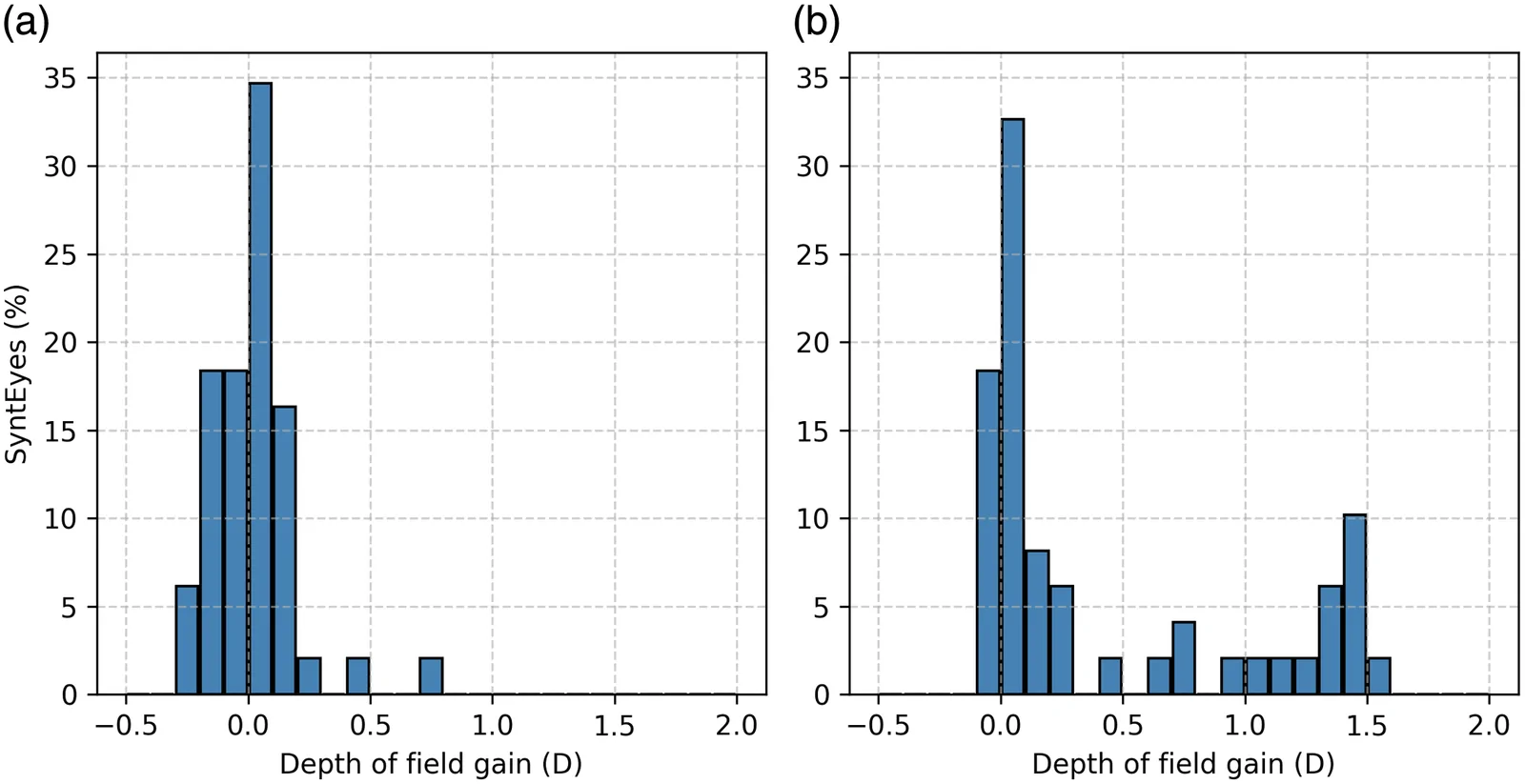
Depth of field gain with use of a freeform surface generated by our model with (a) a 3 mm pupil and (b) a 5 mm pupil.

The depth of field gains (as also defined in Sec. 2.4) are shown in Fig. 9. For small pupil size, the freeform surfaces generated by our model changed the depth of field, compared with a classical EDOF, by 0.00±0.18 D, with a decreasing DOF up to −0.3 D for 41% of SyntEyes, and an increasing DOF up to 0.8 D (bust mostly≤0.2 D) for 59% of them. This slight reduction of the depth of field seems to be coherent with the explanation given previously about the improving values of the peak values. For large pupil size, 91% of SyntEyes increased their depth of field up to 1.7 D (72% with L2), and 9% of them have a reduction of their depth of field up to  −0.1 D (28% up to −0.1 D with L2 up to −0.5 D). In terms of depth of field performance, the performances of the freeform surfaces generated by our model are better than achieved by the theoretical freeform surfaces (L2), which is coherent because there are still some uncorrected aberrations due to the difference between L2 and L3, slightly improving depth of field.

Tables summarizing the performance of the different lenses and the percentages of eyes that showed an improved or reduced DOF are, respectively, shown in Tables 4 and 5.

**Table 4:** Performances of different IOLs studied. L1, classic EDOF. L2, EDOF with a freeform surface calculated by Zemax. L3, EDOF with a freeform surface generated by our model.

	Refractive error (D)			DOF change compared with L1 (D)		Max value VSOTF		
Pupil	L1	L2	L3	L2	L3	L1	L2	L3
3 mm	−0.39±0.32	−0.15±0.09	−0.21±0.12	0.06±0.16	0.00±0.18	0.248±0.044	0.278±0.039	0.298±0.048
5 mm	−0.05±0.32	−0.04±0.06	0.03±0.03	0.33±0.55	0.42±0.58	0.154±0.050	0.318±0.044	0.294±0.041
Difference between large and small pupils	0.34±0.47	0.11±0.09	0.25±0.11					

**Table 5:** Percentage of Synteyes with depth of field that has been degraded or improved thanks to a freeform surface.

Pupil		SyntEyes with decreasing DOF	SyntEyes with increasing DOF
3 mm	L2	34% up to -0.3 D	66% up to 0.5 D
	L3	41% up to -0.3 D	59% up to 0.8 D
5 mm	L2	28% up to -0.5 D	72% up to 1.7 D
	L3	9% up to -0.1 D	91% up to 1.6 D

## 4 Tolerancing

Two main factors may degrade the performance of freeform intraocular lenses (IOLs). The first relates to the freeform surface itself, which may deviate from the intended geometry due to manufacturing tolerances. As the targeted freeform corrections are designed to compensate for corneal aberrations with magnitudes comparable to spherical aberration, which is already routinely corrected by commercially available IOLs, this factor was not addressed in the present study. The second factor concerns the positioning of the freeform IOL within the eye, which may differ from the configuration for which the surface was originally optimized. In this part, the robustness of freeform IOLs generated by the model to position errors, namely, decentration and rotation, was assessed. The EDOF component was removed from the freeform IOLs during the tolerancing analysis to isolate and quantify the ability of the freeform component alone to correct for ocular aberrations. Indeed, the tolerancing of an EDOF system is a well-known topic that we did not wish to revisit. Our sole objective was to analyze the sensitivity introduced by the freeform aspect, independently of the EDOF technique used. Thus, for each SyntEye corrected by a freeform IOL, the EDOF component was first removed (Table 5).

For each tolerancing parameter, we evaluated the impact on the peak monochromatic VSOTF (for computational efficiency) as well as on the refractive error, for a 5 mm entrance pupil diameter. To determine the threshold beyond which the freeform IOL no longer provides sufficient correction, a visual acuity criterion of 0.0 logMAR was adopted, corresponding to a monochromatic VSOTF value of ∼0.32.^27^

### 4.1 Decentration

**Fig. 10:**
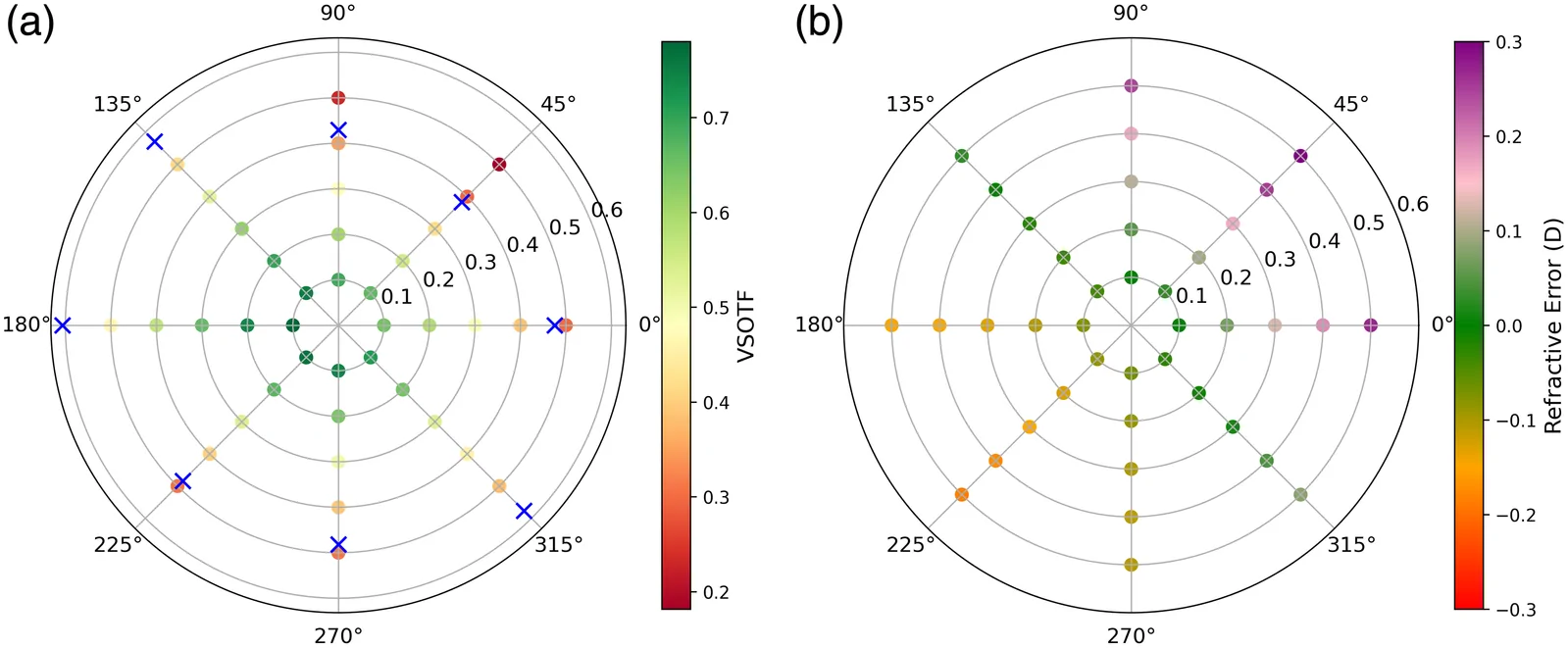
(a) VSOTF achieved by one SyntEye corrected with a freeform IOL, depending on its axial misalignment in mm. Blue crosses represent the misalignment where VSOTF=0.32. (b) Impact on the refractive error due to the misalignment. Each point represents the center of the IOL relative to its original position.

**Fig. 11:**
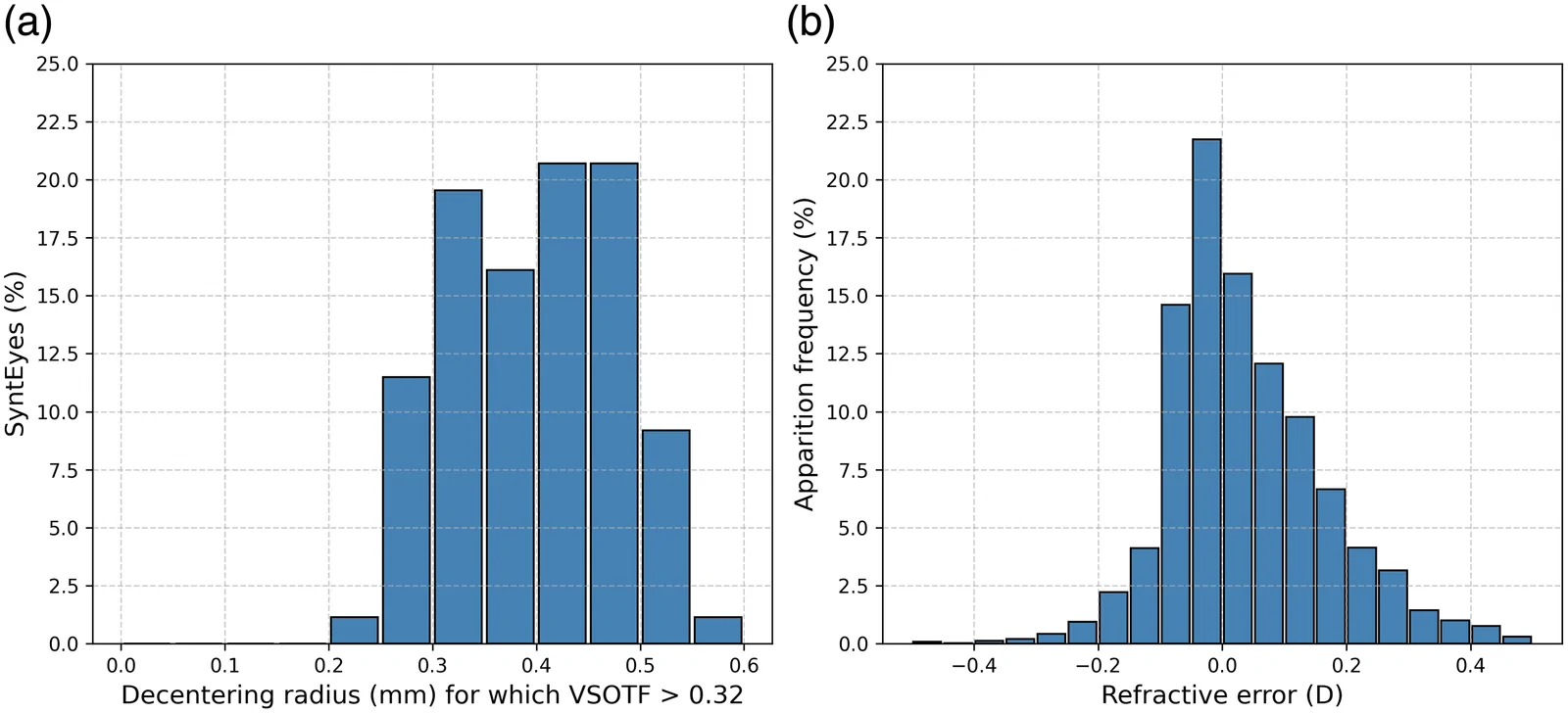
(a) Distribution of the freeform IOL decentration radii for which the VSOTF is better than 0.32, (b) Apparition frequency of refractive error induced by a misalignment (r, θ) of the freeform IOL, for all SyntEyes, with r ranging from 0.1 to 0.5 mm in 0.1 mm steps, and θ ranging from 0 deg to 315 deg in 45 deg steps.

To assess robustness to decentration, each IOL was transversely decentered relative to their original position by a radial distance r in a direction θ, with r ranging from 0.1 to 0.5 mm in 0.1 mm increments, and θ ranging from 0 deg to 315 deg in 45 deg steps. The monochromatic VSOTF of a given SyntEye as a function of IOL decentration is shown in Fig. 10 for illustration, where each point represents the location of the IOL center. For each angle θ, a second-order polynomial fit was applied to estimate the radial distance at which the VSOTF drops to 0.32. These radial values were then averaged over the eight angular directions to obtain a theoretical decentration tolerance for which the freeform IOL maintains adequate correction of the SyntEye. For refractive errors, the sign of the induced refractive error depends on the angle of the decentration, and its absolute value may increase with a radius of misalignment. Instead of calculating the average refractive error induced by an IOL misalignment (which was close to zero), we calculated the apparition frequency of refractive error among all configurations. The results for all SyntEyes are presented in Fig. 11. Figure 11(a) shows the distribution of the decentering radius for which the VSOTF of SyntEyes is better than 0.32. The resulting mean radius was r=0.397±0.081  mm. A study published in 2023 by Langenbucher et al.^32^ demonstrated that the transverse positioning of an IOL can be predicted with an accuracy of ±0.265  mm (or in an area of $∼0.22\;{\text{mm}}^{2}$), which is smaller than the decentration tolerance for 94% of the freeform IOLs. Figure 11(b) represents refractive error results, showing that 63% of configurations induce a refractive error between ±0.1 D, and 91.5% between ±0.25 D, which is clinically robust. The induced refractive error is globally shifting toward a positive value. These results show that despite clinical tolerances on the IOL alignment, a freeform IOL gives robust performance on the best visual acuity and refractive error.

### 4.2 Clocking

To evaluate rotational robustness, each freeform IOL was rotated around its optical axis over a range from −20  deg to +20  deg. For each SyntEye, the relationship between VSOTF and rotational misalignment was fitted to determine the rotation angle corresponding to a VSOTF value of 0.32.

**Fig. 12:**
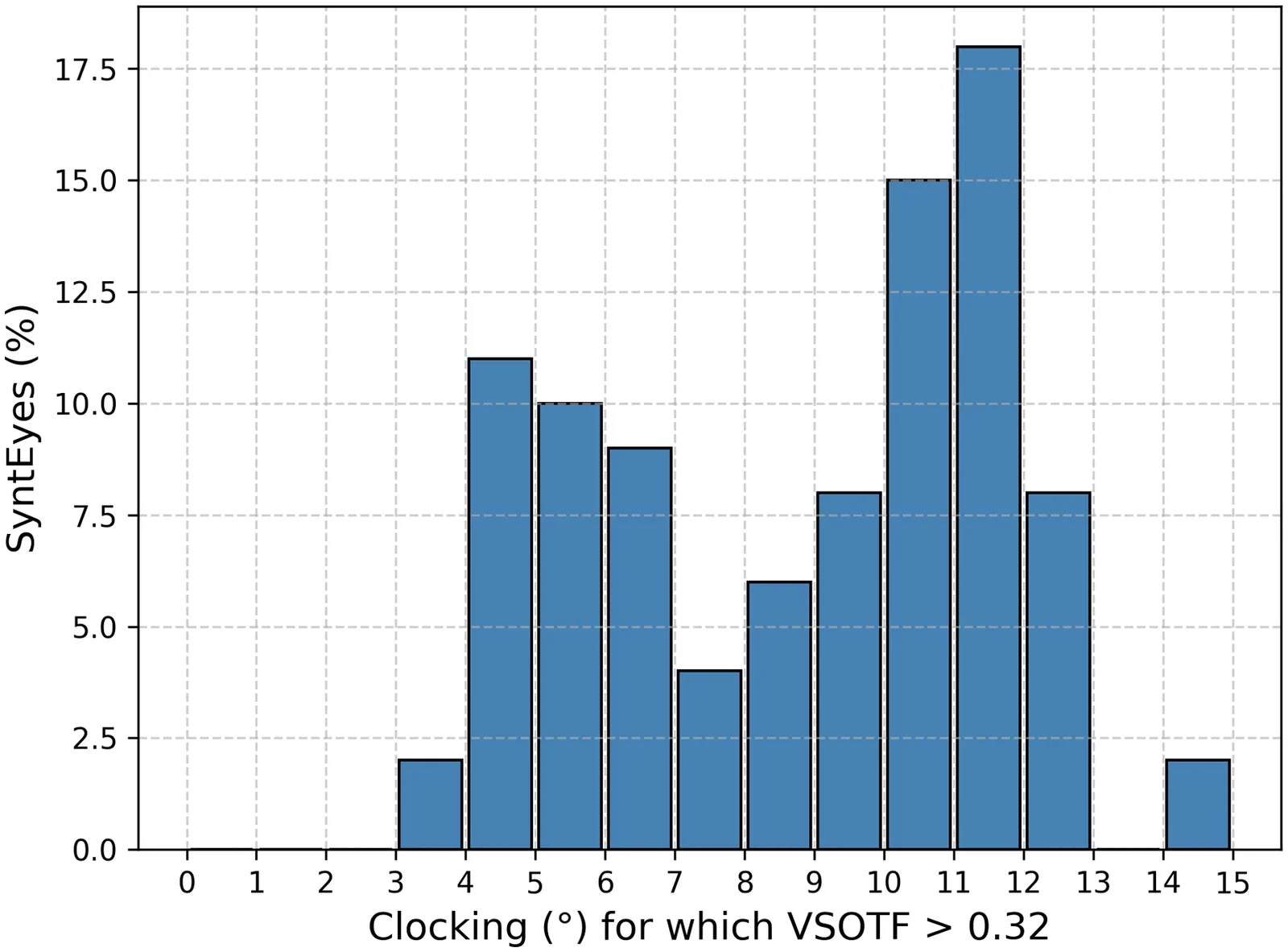
Distribution of the freeform IOL clocking for which VSOTF is better than 0.32.

The resulting distribution of tolerable IOL rotation is presented in Fig. 12. Overall, 87% of the freeform IOLs demonstrated rotational robustness exceeding 5 deg. This finding is encouraging regarding reported clinical outcomes, where post-operative rotation remained below 5 deg in 91.9% of eyes implanted with the AcrySof Toric IOL (n=626) and in 81.8% of eyes implanted with the TECNIS Toric IOL (n=647).^33^

### 4.3 Tilt

To evaluate robustness to tilt, freeform IOLs were tilted relative to their original position with a magnitude tilt ranging from 2 deg to 10 deg in a 2 deg step, and with a direction ranging from 0 deg to 315 deg in 45 deg step. As previously done in Sec. 4.1, the relationship between VSOTF and tilt magnitude was fitted for each direction to determine the magnitude corresponding to a VSOTF value of 0.32. These magnitude values were then averaged over the eight angular directions to obtain a theoretical tilt tolerance for which the freeform IOL maintains adequate correction of the SyntEye. For refractive errors, to unbiased the apparition frequency of refractive error, only tilts of 4 deg or less were included, as 100% of the prediction tilt errors in the study of Langenbucher et al.^32^ are within ±3  deg.

**Fig. 13:**
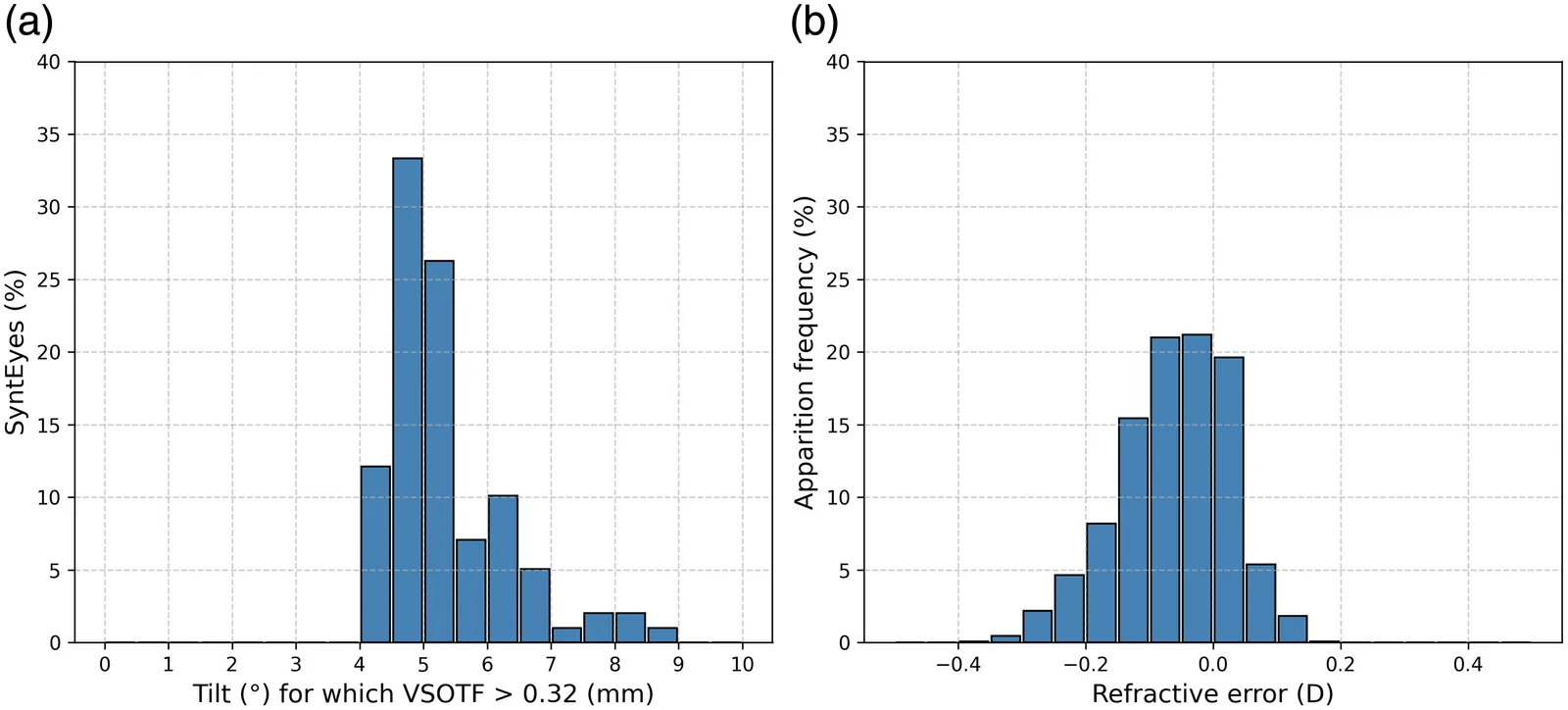
(a) Distribution of the freeform IOL average tilt for which VSOTF is better than 0.32. (b) Apparition frequency of refractive error induced by an IOL tilt of 2 deg or 4 deg in every direction.

The results are summarized in Fig. 13 for all SyntEyes, with (a) the distribution of tilt magnitude for which the monochromatic VSOTF still remains greater than 0.32 and (b) the apparition frequency of refractive errors with tilt magnitude equal or less than 4 deg. As all freeform IOLs demonstrate tilt robustness exceeding 4 deg for the VSOTF criterion, and the recent model achieves an IOL tilt prediction error of ∼1.5  deg, we conclude that freeform IOLs are clinically robust against tilt regarding best visual acuity. Likewise, with all SyntEyes, 92% of configurations with tilt error equal or less than 4 deg induced a refractive error within ±0.20 D, which is clinically robust.

## 5 Discussion and Conclusion

In this study, we investigated the contribution of a freeform surface to an EDOF IOL on the robustness of post-operative performances in the face of individual variations in biometric parameters under polychromatic conditions for small (3 mm) and large (5 mm) pupil size. To do this, we used a database of synthetic eyes, adapted it to simulate pseudophakic eyes, then estimated post-operative performance (refractive error and depth of field) using polychromatic VSOTF. We showed that a freeform surface calculated using preoperative data (HOA of the cornea, ELP predicted by preoperative data, ELP-retina distance, and transverse position of the crystalline lens) would, on average, reduce the difference between the target refraction and subjective refraction (based on the VSOTF), for both small and large pupil sizes. In addition, it would significantly reduce refractive error variations induced by individual biometric variations such as corneal aberrations and transverse IOL positioning. The variation of the refractive error between pupil size was also reduced thanks to the surface freeform.

The addition of the freeform surface also improved the depth of field, mainly with large pupil size, which is not surprising due to the greater weight of higher-order aberrations.

This study shows the potential of freeform optics to improve the performance of EDOF IOLs, but a number of limitations should be considered. First, if globally with the proposed method, the DOF is not affected or increased (72% for large and 66% for small pupil), it is slightly reduced in several eyes (28% for large and 34% for small pupil). This suggests that even if the proposed method generally allows for the improvement of the performance of a conventional EDOF IOL, there are still avenues for improvement. In addition, the model was trained on a database of nonreal eyes, which was derived from measurements of 312 real eyes. The physiological parameters are therefore potentially biased. As the linear regression model is highly dependent on the training database, it is possible that it may generate a freeform surface that is not adapted for an eye outside of this training database. For example, the maximum IOL decentration values here are around 0.6 mm, whereas some studies have reported values greater than $0.6\;{\text{mm}}^{1}$.

Second, the model generates freeform surfaces using preoperative physiological parameters, but it is necessary to keep in mind that some assumptions have been made. Changes in these parameters after surgery are limitations for the method proposed here. As a reminder, these parameters are corneal HOA, ELP, and lens decentration. We supposed here that the HOA of the cornea is not changed by the surgery. This hypothesis is coherent as corneal HOA varies slightly during a micro-incision,^17^ but a model for predicting post-operative HOA may be necessary for the use of freeform optics on IOLs. About the ELP, its prediction is already a current challenge for power calculation. However, even if a longitudinal change in IOL positioning certainly has an impact on refractive error, it has a minor one on the capability of the freeform surface to correct the aberrations of the patient’s eye (this of course is different for the transverse position of the IOL which had to be considered). Furthermore, the lateral positioning of the IOL is the parameter with the highest risk of unpredictability. Here, we supposed that the crystalline lens and the IOL have the same location. To improve the method, the true transverse position of the IOL must be predicted, and it depends on the haptics design. Nevertheless, this issue seems to be increasingly well handled, as shown by a recent study^32^ predicting IOL decentration, showing a predictability error of ∼±0.265  mm on the centering of the IOL, and freeform IOL generated in this study is robust to a decentration error of 0.397±0.081  mm. Furthermore, the robustness analysis of the freeform IOLs showed that 87% of the designs maintained stable optical performance, considering both the monochromatic VSOTF peak and refractive error, in the presence of rotational misalignment ranging from 5 deg to 15 deg, whereas clinical rotation is globally inferior than 5 deg.^33^ Similarly, regarding tilt robustness, the results show sufficient robustness for tilt errors greater than 4 deg, whereas a new model achieves a 1.5 deg error prediction. Such tolerance indicates a clinically relevant level of rotational robustness.

In addition, the freeform surface added to the EDOF IOL is designed here without the EDOF “performance part.” It would have been entirely possible to include it in the optimization, but the method proposed here would then have been dependent on the EDOF technology used, and we wanted to propose a method that could be applied regardless of the EDOF IOL.

Designing an implant is a complex process, and additional studies are necessary to define the most effective freeform profile while taking into account machining constraints. This study, however, highlights the value of the freeform approach, which makes it possible to reduce the impact of individual variations in biometric parameters, both for small and large pupil sizes, and proposes a first concrete method to achieve this.

## Data Availability

As stated in Sec. 2, this study used the database proposed by Rozema et al.,^14^ which is distributed under a CC-BY-NC-ND 4.0 International License. Limited data and code were generated otherwise, and these materials are available from the corresponding author upon reasonable request.
